# Impact of celebrity disclosure on mental health-related stigma

**DOI:** 10.1017/S2045796022000488

**Published:** 2022-08-30

**Authors:** Petra C. Gronholm, Graham Thornicroft

**Affiliations:** Centre for Global Mental Health and Centre for Implementation Science, Health Service and Population Research Department, King's College London, Institute of Psychiatry, Psychology and Neuroscience, London, UK

**Keywords:** Celebrity, disclosure, discrimination, stigma

## Abstract

Mental health stigma and discrimination are global problems, and their reduction is recognised as an important public health priority. Involving celebrities in stigma reduction is increasingly common. This Editorial considers the impact of celebrity disclosure on mental health-related stigma; that is, whether and how a famous person openly speaking about their experience of mental health conditions can reduce stigma. Potential explanations for how celebrity mental health disclosures can influence mental health-related knowledge, attitudes and behaviours are outlined, followed by an overview of evidence on how celebrity disclosure operates to reduce stigma. Considering the available evidence, we provide a number of conclusions and recommendations for how celebrities can effectively be involved in anti-stigma efforts, and what considerations this requires. It is fair to say that celebrity disclosures can support stigma-reduction efforts through increasing the public's awareness of mental health, modelling behaviour and generating openness on speaking about mental health problems, and on seeking help when needed. However, whether celebrity disclosure achieves changes in mental health stigma-related knowledge, attitudes and behaviours depends on the extent to which there is a match between the attributes of the famous person, the content shared in their disclosure narrative and the intended audience of the message. Further research is needed on all these questions to better understand how to successfully utilise the potentially huge power of celebrity disclosure in large-scale anti-stigma efforts.

## Introduction

Stigmatisation refers to the devaluation and discrimination expressed towards, and experienced by, people with a mental health condition (Link and Phelan, 2001; Thornicroft *et al*., 2007). Mental health stigma and discrimination are global problems (Thornicroft *et al*., 2009; Lasalvia *et al*., 2013) associated with a range of negative consequences and adverse impacts on wellbeing in a number of ways, including worsening of psychological distress, inhibition of help-seeking and treatment adherence, poor access to mental and physical healthcare, reduced life expectancy, limiting of personal relationships and reductions in the ability to achieve educational and vocational goals (Reavley and Jorm, 2015; Gronholm *et al*., 2017). For many people, these consequences have been described as worse than the experience of the mental health condition itself (Thornicroft, 2006; Thornicroft *et al*., 2016). Reducing stigma and discrimination is recognised as an important public health priority (WHO, 2013; Hoffner and Cohen, 2018; Thornicroft and Sunkel, 2020).

Involving celebrities in stigma-reduction efforts is increasingly common. Specifically, celebrities' disclosures of mental health conditions are seen as potentially positively influencing health behaviours such as help-seeking and changing public attitudes towards people with mental health conditions (Corrigan *et al*., 2014; Lee, 2019; Francis, 2021). In this Editorial we discuss this type of celebrity disclosure; that is, when a famous person openly speaks out about their experience of mental health conditions, and the impact of this on stigma reduction.

In recent years, a number of well-known people globally have made such disclosures, including television and film actors, musicians, politicians and athletes. This includes, for example, internationally famous singers Lady Gaga, Mariah Carey and Adele, tennis legend Serena Williams, celebrated gymnast Simone Biles, Hollywood and Bollywood films stars such as Dwayne ‘The Rock’ Johnson and Deepika Padukone, TV stars Oprah Winfrey and Nana Kinomi and celebrated writers such as Chimamanda Adichie. The age of social media has introduced another type of celebrity famous via their online platforms – influencers, vloggers, live streamers and other internet personalities. Disclosures of mental health conditions have also happened among such celebrities, for example, the YouTuber Zoella, and TikTok stars Charli D'Amelio and Addison Rae.

In addition to serendipitous mental health disclosures that are picked up by news and media (Van Beveren *et al*., 2020), celebrity disclosure stories have also been strategically embedded within structured anti-stigma programmes. Such efforts can range from small-scale local workshops with elements of ‘celebrity examples’ (Jones *et al*., 2011; Gibson *et al*., 2019) to large-scale national campaigns. For example Time to Change in England (Eaton, 2009) and Like Minds Like Mine in New Zealand (Vaughan and Hansen, 2004) included media advertisements involving stories of celebrities (e.g. politicians, TV personalities and actors) who have experienced mental health conditions.

Reflections of people affected by stigma have indicated that media portrayals of mental ill health and celebrity disclosures can be perceived to have an impact on the general acceptance of people with mental health conditions and can reduce public stigma (Chung *et al*., 2019). Also, evaluations of anti-stigma interventions and campaigns involving celebrity stories show that they are generally seen to be effective, but there has been no particular attention to the mechanisms, or perceived impacts of specific celebrity disclosures. We are bringing focus to this area of research through this Editorial by consolidating current theories and evidence on the impact of celebrity disclosure on stigma reduction and what we can conclude from this, to guide how anti-stigma programmes and other stigma reduction strategies can best harness the power of celebrity in their efforts.

## How can celebrity disclosure reduce stigma?

There are a number of potential explanations for how celebrity mental health disclosures can influence mental health-related knowledge, attitudes and behaviours. One pathway is by raising awareness and understanding. Celebrity disclosure can bring positive attention to a health condition (Ferrari, 2016), leading to more positive health behaviours such as sourcing reliable information and help-seeking. (Chung *et al*., 2019; Lee, 2019; Leung, 2019).

Celebrity disclosure can also serve an educational role, increasing public knowledge about symptoms, treatments and services, and correcting misconceptions regarding mental health and mental illness (Beck *et al*., 2014; Lee, 2019; Francis, 2021). It is arguable that increased awareness, understanding and interpersonal communication can decrease both public (inter-personal) stigma and self-stigma (Corrigan *et al*., 2014; Francis, 2018; Hoffner and Cohen, 2018; Lee, 2019; Corrigan *et al*., 2022). Seeing an admired public figure speaking openly about mental health issues could also reduce stereotypes and normalise mental health conditions (Hoffner and Cohen, 2018), which can further reduce stigma.

Celebrity disclosure can also lead to stigma change through social modelling. Specifically (according to social learning theory principles), audiences may learn behaviours from celebrities through observing their behaviours and their consequences (Lee, 2019). Seeing that a celebrity can experience and disclose mental health issues, and still be considered attractive, admired and in good social standing can dispel fears of the potential negative consequences of disclosure (Lee, 2019). Thus seeing celebrities speak out about their mental health can challenge the norm of concealing mental illness and can encourage help-seeking (Leung, 2019).

Exposure to mass media reporting of celebrity disclosures and celebrity mental health content has been associated with openness about such topics in online conversations (Francis, 2021). However, research about celebrity disclosures has generally focused on exploring specific outcomes such as information or help-seeking, with much less attention given to evaluating actual stigma change.

Whether a person will be influenced by a celebrity's disclosure also depends on how they relate to the particular celebrity. One such mechanism is having a parasocial relationship with the celebrity. This refers to a person's one-sided perceived interpersonal relationship and emotional connection with a distant figure, often a media figure or other type of celebrity. Familiarity with famous people through frequent media (including social media) exposure can lead to a sense of knowing them or even having a deep emotional attachment (Hoffner and Cohen, 2018; Lee *et al*., 2021). In this respect such a parasocial relationship with a celebrity can be considered similar to other close social affiliations. Such connections can have an emotional significance and value, and an influence on a persons' beliefs, attitudes and behaviours. Indeed, contact with members of a stigmatised group – whether direct or indirect parasocial contact – is an effective stigma-reduction strategy (Gronholm *et al*., 2017; Hoffner and Cohen, 2018; Maunder and White, 2019).

Further, how far a person identifies with a given celebrity can influence the impact of their mental health disclosure, through influencing whether and how a person internalises the celebrity's attitudes, values and beliefs, and then adopts their behaviours (Francis, 2018; Lee, 2019; Lee *et al*., 2021). Such identification with another person can occur in two ways – vertical and horizontal (Lee, 2019). In vertical identification a person identifies with someone considered to be superior to them, or someone they admire, such as a famous person. In horizontal identification people are considered to identify more strongly with others, including celebrities, who possess similar characteristics to themselves, or example in terms of age, gender or other circumstances.

This sense of perceived similarity with a celebrity may be important when considering the impact of their mental health disclosure. Although celebrities can bring positive attention and increased awareness and education about mental health issues (Ferrari, 2016), it is also possible that famous people are considered too dissimilar from ‘ordinary’ people with mental health conditions, and too protected from the challenges of everyday life, for their story and experience to resonate and have a positive impact (Corrigan *et al*., 2022). This reflects the process of ‘subtyping’, where a stereotyped group (e.g. people with mental health conditions) is separated into subgroups where some are thought to confirm with the stereotype (here; ‘ordinary’ people) whereas others are considered atypical and exempt from judgement (here; celebrities) (Bott and Murphy, 2007). Promoting acceptance and inclusion by focusing only on the atypical subgroup can counterintuitively perpetuate or even increase negative stereotyping, as it does not challenge the negative perceptions of the group as a whole (Hakim *et al*., 2020). Source credibility and authenticity is another key factor regarding the influence of celebrity disclosure. Stories from sources perceived to reflect expertise and trustworthiness are more credible and have a greater impact (Leung, 2019). Celebrity disclosure can signal authenticity, as sharing experiences regarding a stigmatised mental health condition can be seen as real, showing vulnerability, and as providing access to a celebrity's inner, personal life in contrast to a carefully curated public persona. Thus, disclosure can make a celebrity seem more authentic, relatable and therefore more credible, so making their disclosure more impactful (Lee *et al*., 2021).

## Evidence of how celebrity disclosure operates to reduce stigma

Despite such theoretical approaches to how celebrity disclosures can lead to stigma change, not many studies have examined these processes and whether celebrity disclosure actually reduces stigma.

Corrigan *et al.* examined the value of fame in stigma change by comparing the impact of disclosure vignettes from a celebrity *v.* a non-celebrity (Corrigan *et al*., 2022). The celebrity vignette came from US pop singer Mariah Carey who has spoken out about living with bipolar disorder, and the non-celebrity vignette was selected from a published compendium of recovery narratives which describe living with depression and schizophrenia (Corrigan *et al*., 2015). The vignettes were matched on gender, race and age. The study found that participants perceived the non-celebrity as more similar to them and more likeable than the celebrity. Reading the non-celebrity vignette led to a greater reduction in reported stigma compared to the celebrity vignette. The authors concluded that disclosure stories from non-celebrities might hold more power in reducing public stigma than celebrity disclosures, although celebrities speaking out may still support stigma reduction through increasing public awareness of mental health which can lead to more positive responses to mental health challenges of others, and in modelling behaviour to be more ready to speak out.

Slightly different findings on the impact of the level of perceived fame and celebrity were reported by a study which examined how media coverage of celebrity disclosure of panic disorder influenced public health behaviours such as seeking information and care (Lee, 2019). The study found a positive correlation between media coverage of celebrity disclosures and frequency on searching, asking and providing information. This effect was larger when the celebrity was more famous. It was concluded that celebrities' disclosures hold a positive role in reducing societal stigma and negative attitudes towards panic disorder, and that this impact could also likely be found for other types of mental health condition.

Hoffner and Cohen explored whether having a parasocial relationship with a celebrity (specifically, actor Robin Williams) influenced reactions following the news of his death by suicide (Hoffner and Cohen, 2018). They found that among people with a stronger parasocial relationship there was less stigma towards people with mental health conditions, with less desire for social distance from people with depression, more willingness to personally seek treatment if they experienced depression within the following year and more frequently reaching out to support others.

This is similar to the finding of a study examining celebrity–fan relationships. This study found that having a favourable celebrity–fan relationship was positively correlated with awareness of depression following media stories of celebrity depression disclosures (Leung, 2019). In this case, a stronger relational bond between audience and celebrity resulted in a stronger positive reaction to media stories of disclosure.

The influence of parasocial relationships and the perceived similarity with a celebrity was also examined in a study focused on ‘microcelebrities’ (Lee *et al*., 2021) – online streamers with many followers on platforms like YouTube and Twitch, and who can be viewed as more authentic, real, relatable, engaging and transparent than ‘traditional’ celebrities. This study found that depression disclosures increased the perceived authenticity of the microcelebrities, but not their credibility. Having a parasocial relationship with a microcelebrity was linked to their perceived credibility, but identifying with the celebrity was not. Consequently, understanding the type of relationship a viewer has with a microcelebrity can help to understand how their disclosures can provide opportunities for health awareness and other interventions. This paper presented the intriguing idea that even if online microcelebrities are not considered to be a credible source of knowledge, they can still help reduce social stigma around opening up about mental health problems.

Qualitative studies have provided interesting insights into the complexity of perceived credibility of celebrity disclosure. A study on the impact of celebrity disclosures of a diagnosis of major depressive disorder found mixed reactions. Some respondents reported more positive attitudes towards such celebrities following the disclosure, whereas others reacted with pity, or doubted whether the story was genuine or simply used to gain publicity (Leung, 2019). The study considering microcelebrities also noted that some viewed celebrities' disclosure of depression as a strategic decision to gain attention, leading to questions regarding their credibility (Lee *et al*., 2021). There have also been mixed reactions to whether a celebrity disclosure is self-disclosed or ‘revealed’ by the media. Some considered self-disclosed stories more credible than when diagnoses were revealed by the media, whereas others felt self-disclosure was a publicity-seeking act and that media disclosure was more trustworthy (Leung, 2019).

## What can we conclude about celebrity disclosure and stigma?

From this overview of relevant publications, and their inconsistent findings, we would cautiously conclude that:

1. Celebrities with personal experience about the condition of which they speak are likely to be more impactful messengers, as celebrities' direct experiences with mental health issues increases their authenticity and perceived credibility (Lee *et al*., 2021). This also suggests celebrities simply endorsing mental health messages is not likely to be as impactful as opening up about personal experiences.
2. Disclosure stories by famous people who despite their celebrity status are perceived to be similar to the target audience in some respects are more likely to lead to positive stigma change (Corrigan *et al*., 2022).
3. It is necessary to consider the nature of the media portrayals and how members of the public psychologically process the content to realise the potential of celebrity disclosure to reduce stigma (Hoffner and Cohen, 2018). For example, celebrity disclosure of mental health problems through media stories is not in itself sufficient as it can remain superficial and fuel hearsay and gossip. Rather, reporting on celebrities' disclosures should also provide solid information about the cause of the condition and about effective treatments (Leung, 2019).
4. Given the power of media coverage of serendipitous celebrity disclosures, we propose that more active use is made of the media through, for example, developing specific culturally sensitive messaging, online hashtags and campaigns to increase awareness building on celebrity disclosures (Francis, 2021).
5. The public health community should advocate for continued disclosure by celebrities as it has been shown to have a positive impact on communication and health behaviours. Celebrities who are open about their mental health issues should be recruited, trained and supported to act as social media influencers by sharing destigmatising disclosure stories (Francis, 2021).
6. There should also be more involvement of microcelebrities (Lee *et al*., 2021). Internet and social media have facilitated the creation of strong parasocial relationships with online celebrities. Traditional celebrities are characterised by their privilege and distance from the mundane, whereas microcelebrities may more effectively emphasise their accessibility and authenticity.
7. There is also a clear need for research to examine the causal processes between the public's relationship with celebrities, celebrity disclosure messages and reaction to these in terms of stigma reduction through changes to knowledge, attitudes and behaviours (Francis, 2018, 2021; Hoffner and Cohen, 2018; Lee *et al*., 2021).
8. More research is also needed to understand the impact of celebrity health disclosures on specific groups, in particular minority groups (e.g. Black young males) who are generally underrepresented in research (Francis, 2018).
9. It is important to examine further person-level variables that might interact with fame, for example, the type of fame a person has (e.g. film, music, sports or politics) (Corrigan *et al*., 2022).
10. A specific aspect that needs further research is how the content of the disclosure influences its effect on the public (Lee *et al*., 2021).
11. Further insights are needed regarding potential differences in how celebrity disclosure is most impactful at different levels of reach (e.g. locally, regionally, nationally and internationally).
12. More needs to be understood on whether the medium or channel of disclosure has a specific type of impact and on what socio-demographic social groups (e.g. TikTok, Instagram, YouTube, TV, film or print media).

To conclude, we encourage the continued involvement of celebrities in anti-stigma initiatives but call for increased consideration of the theory and evidence base underpinning how, when and which celebrities are likely to be most efficient in these efforts. It is also important to consider the impact of disclosure stories by ‘ordinary’ people, and when their use would be helpful and appropriate. Through this Editorial we highlight how there are limited systematic evaluations about the effect of celebrity mental illness disclosures on stigma or evidence of stigma change, despite how this phenomenon is growing and is considered to have important implications for anti-stigma strategies and public health education efforts (Francis, 2018; Lee, 2019). Yet it is fair to say that celebrity disclosures can support stigma reduction efforts through increasing the public's awareness of mental health, modelling behaviour and generating openness on speaking about mental health problems, and on seeking help when needed. However, whether celebrity disclosure achieves changes in mental health stigma-related knowledge, attitudes and behaviours depends on the extent to which there is a match between the attributes of the famous person, the content shared in their disclosure narrative and the intended audience of the message. Further research is needed on all these questions to better understand how to successfully utilise the potentially huge power of celebrity disclosure in large-scale anti-stigma efforts.
